# The Ultimate Icebreaker: The *Rhagium mordax* Antifreeze Protein as a Universal Ice Nucleation Inhibitor

**DOI:** 10.3390/polym18212625

**Published:** 2026-10-23

**Authors:** Maria A. Majorina, Vladimir N. Uversky, Bogdan S. Melnik

**Affiliations:** 1 Protein Spectroscopy Group, Institute of Protein Research RAS, Institutskaya 4, 142290 Pushchino, Russia; mariamazhorina@yandex.ru; 2 Department of Molecular Medicine and USF Health Byrd Alzheimer’s Research Institute, Morsani College of Medicine, University of South Florida, Tampa, FL 33612, USA

**Keywords:** antifreeze protein, ice-nucleating, *Rhagium mordax* ice-binding protein, inhibition of ice nucleators

## Abstract

Antifreeze proteins were discovered quite some time ago. The protein from *Rhagium mordax* is considered one of the most active among them. The literature has demonstrated this protein’s ability to bind to the surface of ice, and its effect on the survival of *R. mordax* larvae has also been investigated. In this work, we examined the ability of this protein to inhibit ice nucleators of different origin. As ice nucleators, we selected inactivated *P. syringae* bacteria, silver iodide, and insoluble metal oxide powders, for which we had previously demonstrated the ability to act as ice nucleators at different temperatures. Since the *R. mordax* antifreeze protein blocks all biological and inorganic ice nucleators examined in this study, and taking into account its ability to structure water molecules on its surface, we hypothesized that *R. mordax* antifreeze protein can serve as a universal inhibitor of ice nucleator.

## 1. Introduction 

Antifreeze proteins were discovered quite some time ago and were identified as proteins that help cells or organisms survive at subzero temperatures [1,2,3,4,5,6,7,8]. It was subsequently found that many of these proteins are able to bind to the surface of ice; such proteins were termed ice-binding proteins [2,9,10,11]. Several viewpoints exist in the literature regarding how ice-binding proteins help organisms survive at subzero temperatures. The first proposes that ice-binding proteins bind to the surface of growing ice crystals and arrest or slow their growth [9,12,13,14]. The second proposes that ice-binding proteins affect ice recrystallization: during prolonged incubation of samples at subzero temperatures, large ice crystals grow larger while small crystals shrink; in the presence of antifreeze proteins, the growth of large crystals is arrested (e.g., Refs. [15,16,17]). The third viewpoint proposes that ice-binding proteins block ice nucleators [18,19,20], keeping water in a supercooled, liquid state so that ice does not form and does not damage cells. These three viewpoints are not mutually exclusive: it is thought that some antifreeze proteins are able to arrest ice growth, affect ice recrystallization, and block ice nucleators all at once, whereas for other antifreeze proteins only one of these properties has been demonstrated [2,9,10,11].

**Figure 1:**
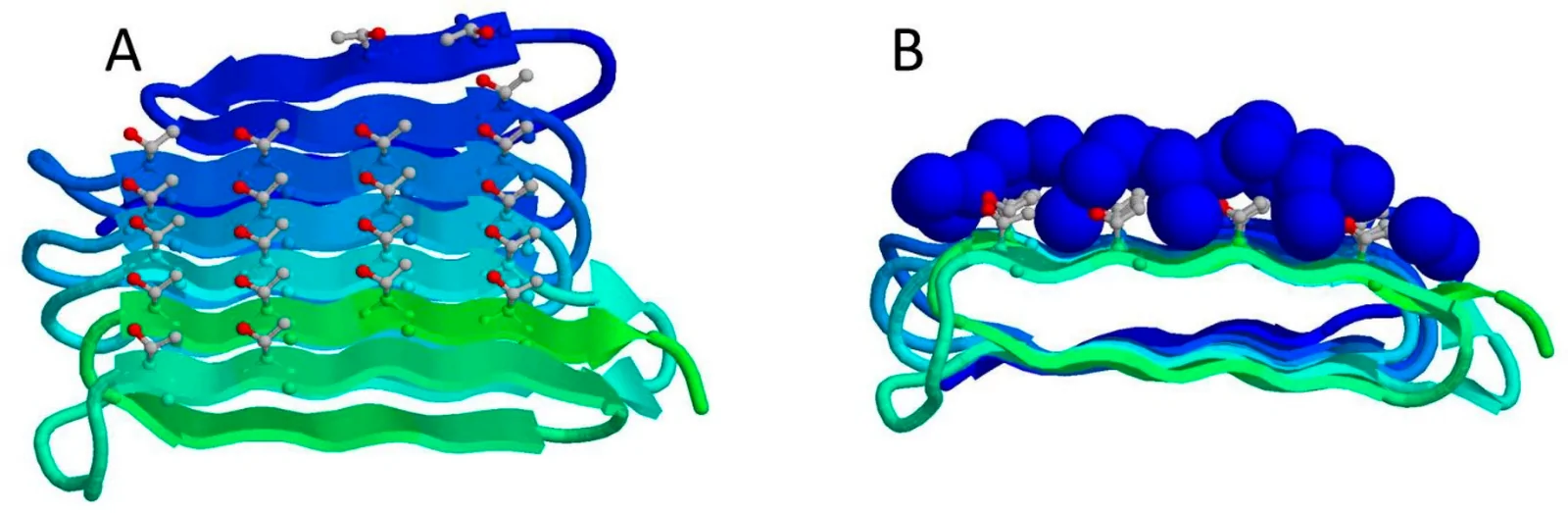
Structure of the antifreeze protein from *Rhagium mordax* (*Rm*AFP), PDB: 6XNR [21]. β-strands of the protein are colored from the N-terminus (blue) to the C-terminus (green). (**A**) View of the ice-binding face of the protein. (**B**) End-on view of the β-sandwich. On the ice-binding surface of the protein, threonine residues are shown as balls and sticks. Water molecules structured on the protein surface are shown as blue spheres.

The antifreeze protein from *Rhagium mordax* (*Rm*AFP) is considered one of the most active antifreeze proteins known to date (e.g., Refs. [2,8]). *Rm*AFP belongs to the class of ice-binding antifreeze proteins. The structure of this protein resembles a β-sandwich, on one face of which lie four rows of threonine residues that are thought to bind to the surface of ice [21]. Figure 1 shows the structure of this protein.

Binding of *Rm*AFP to the surface of ice is indirectly supported by the large number of water molecules structured on its surface, as well as by theoretical studies, for example using molecular dynamics simulations [22,23,24]. In addition, binding to ice has been demonstrated visually in studies of fusion proteins, in which *Rm*AFP (or its homologue, AFP from Rhagium interrogator (RiAFP), which shares a highly conserved sequence and similar structure that allows for direct comparison) fused to a fluorescent protein stains the surface of ice [25,26,27].

The effect of *Rm*AFP on the survival of R. mordax larvae has also been well studied; it is thought that the protein prevents the growth of ice microcrystals and allows the larvae to survive at temperatures down to −20 °C [25,28]. While the preliminary inhibitory effect of *Rm*AFP1 on specific bacterial nucleator has been briefly reported, the detailed evaluation of the capability of this protein to inhibit the activity of the ice nucleators of different nature (both natural and artificial) has not yet been investigated in detail. Testing against various biological and inorganic nucleating agents conducted in this study revealed that this inhibitory effect is a broad physicochemical interaction rather than one specific to *P. syringae*.

The search for effective ice nucleation inhibitors is a vital task with broad scientific and technological implications. Identifying a universal antifreeze protein (AFP) capable of blocking diverse ice nucleators, both biological and inorganic, would drive significant advancements across biology, medicine, and various industrial fields. Such a protein enables the preservation of water in a supercooled state even in the presence of potent crystallization centers. This precise control over the phase transition of water opens up new possibilities for the cryopreservation of cell cultures and organs, as well as the extended storage of food products at subzero temperatures without the damaging effects of ice formation.

## 2. Materials and Methods

### 2.1. Expression of RmAFP in E. coli and Biomass Preparation for Protein Isolation

A plasmid carrying the gene for the antifreeze protein *Rm*AFP was constructed in *E. coli* cells on the basis of the pHUE vector. The genetic construct contained a histidine tag (His-tag). Gene sequences were verified by sequencing. Competent *E. coli* BL21(DE3) cells were transformed with the plasmid using the standard calcium chloride method.

*E. coli* cells were grown in 5 mL of liquid LB medium at 37 °C with continuous shaking to OD_600nm_ = 0.4–0.6. Cells were then pelleted by centrifugation at 10,000× *g* and 4 °C for 10 min on a HERMLE Z326K centrifuge (HERMLE Labortechnik GmbH, Wehingen, Germany). The pellet was resuspended in 500 μL of ice-cold 0.1 M CaCl_2_ and centrifuged on a DLAB D1524R centrifuge (DLab, Beijing, China) at 5000× *g* and 4 °C for 5 min; this procedure was repeated twice. The cell pellet was resuspended in 100 μL of 0.1 M CaCl_2_ and kept on ice for 20 min, after which the target plasmid was added to the competent cell suspension. The tube was incubated on ice for a further 30 min, then placed in a thermostat at 42 °C for 1 min (heat shock), and returned to ice for 10 min. Transformants were selected on the basis of ampicillin resistance; a 50 μL aliquot of the resulting cell suspension was plated on agar containing the antibiotic (200 μg/mL).

To verify protein expression, 1–2 colonies from the Petri dish were transferred into 5 mL of liquid LB medium containing 200 μg/mL ampicillin and grown overnight at 37 °C and the mixing speed 200 rpm on a MIULAB ES-60 shaker-incubator (Miulab, Hangzhou, China). A 100 μL aliquot of the overnight culture was transferred into 5 mL of LB medium containing 200 μg/mL ampicillin and grown to OD_600_ = 0.5, at which point 0.5 mM of the inducer isopropyl β-D-1-thiogalactopyranoside (IPTG) was added. After induction, the cell culture was grown at 37 °C to OD_600_ = 1.3–1.5. The presence of the antifreeze protein in the cells before and after IPTG induction was verified by SDS-PAGE in a 15% polyacrylamide gel (Supplementary Materials Figure S1).

After overnight incubation at 37 °C, several colonies from the Petri dish were transferred into 100 mL of liquid LB medium containing 200 μg/mL antibiotic and grown overnight at 37 °C on an Excella E24 (New Brunswick, NJ, USA) shaker-incubator. Next, 10 mL of the overnight culture was transferred into 0.5 L of LB medium containing 200 μg/mL antibiotic and grown to OD_600_ = 0.5, at which point 0.5 mM IPTG was added. The cell culture was grown to OD_600_ = 1.3–1.5 at 37 °C. Once the culture reached the required optical density, cells were pelleted on a Beckman J2-21(Beckman Instruments, Fullerton, CA, USA) floor-standing centrifuge at 8000× *g* for 25 min at 4 °C.

*Pseudomonas syringae* strain (*Pseudomonas syringae* pv*.syringae* strain 3023) was cultured in liquid LB medium at 26 °C at under constant agitation at 200 rpm until reaching the late exponential phase. Cells were harvested by centrifugation (HERMLE Labortechnik Z326K, Wehingen, Germany) at 10,000× *g* for 20 min at 4 °C. The supernatant was discarded and resuspended in buffer 10 mM Tris-HCl, 0.1 M NaCl, pH 7.26. To remove residual medium, cells were pelleted and resuspended again in the same buffer; this procedure was repeated twice. In standard experiments, the resulting *P. syringae* suspension in 10 mM Tris-HCl, 0.1 M NaCl, pH 7.26 was used.

### 2.2. Purification of RmAFP

The cell pellet was resuspended in buffer (20 mM Tris-HCl, 0.15 M NaCl, pH 7.8). *E. coli* cell walls were disrupted using an EmulsiFlex-C3 high-pressure homogenizer and centrifuged for 40 min at 30,000× *g* and 4 °C. The supernatant was passed through a 0.22 μm Corning filter and loaded onto a column for affinity chromatography. A HisTrap column (Chelating Sepharose HP IMAC resin, 5 mL bed volume) was equilibrated with buffer containing 20 mM Tris-HCl, 0.15 M NaCl (pH 7.8). The elution buffer had the same composition, supplemented with 0.5 M imidazole (0.5 M imidazole, 20 mM Tris-HCl, 0.15 M NaCl, pH 8). The protein was eluted from the column using a stepwise imidazole gradient; the target protein eluted at 40% elution buffer.

Fractions containing the target protein were transferred, using a PD-10 desalting column (Sephadex G-25 resin), into buffer containing 20 mM Tris-HCl, 0.15 M NaCl (pH 8) for size-exclusion chromatography on a HiLoad 26/600 Superdex 75 pg column (320 mL bed volume) equilibrated with the same buffer.

To remove the histidine tag, the pure fractions obtained after gel filtration were incubated with Usp2 protease for 1.5 h at 37 °C (1 μL of protease per 100 μL of protein solution). After incubation, the protein solution was again loaded onto a HisTrap column (Chelating Sepharose HP IMAC resin, 5 mL bed volume) equilibrated with buffer containing 20 mM Tris-HCl, 0.15 M NaCl (pH 7.2). The elution buffer had the same composition, supplemented with 0.5 M imidazole (0.5 M imidazole, 20 mM Tris-HCl, 0.15 M NaCl, pH 7.2). The target protein, having lost its affinity for the resin, did not bind to the column and eluted in the starting buffer. Purity of the preparations at each purification step was assessed by SDS-PAGE in a 15% polyacrylamide gel (see Supplementary Materials Figure S1). The protein was characterized further using mass spectrometry using a high-resolution Orbitrap Q Exactive HF mass spectrometer (Thermo Fisher Scientific, Waltham, MA, USA).

Prior to the experiments, the protein solution was purified using a dialysis membrane with a molecular weight cut-off (MWCO) of 10 kDa. Dialysis was carried out for a total dialysis time of 12 h at 4 °C, with a total of 3 fluid changes to effectively remove residual salts and imidazole.

The final protein concentration in the samples was 0.02 mg/mL.

### 2.3. Determination of Protein Concentration

Protein concentration was determined using the Waddell method [29] based on the absorbance difference between 215 nm and 225 nm (Conc. = (A215 − A225) × 0.144 mg/mL). The optical path length was maintained at 1 mm using a standard quartz cuvette. A blank correction was performed using the final dialysis buffer, and repeatability was ensured by analyzing all samples in triplicate (relative standard deviation < 10%). The protein concentration was determined in stock solutions, in which the protein was at a concentration of about 0.7 mg/mL in water. Here, the protein concentration was measured after the protein was transferred to water to ensure that the Tris buffer did not interfere with the spectrophotometric measurements. The concentration of such solutions was measured spectrophotometrically. The final experimental concentration of ~0.02 mg/mL was prepared by diluting 28.5 µL of this stock solution into 1 mL of the final sample (water containing the nucleator).

### 2.4. Sample Preparation

Deionized water was used to prepare all samples; all samples had a volume of 1 mL. The low-molecular-weight nucleators AgI, CuO, ZnO, and SnO_2_ are insoluble heterodisperse powders, which were weighed and added at 4–5 mg per sample. Metal oxides were preheated in a furnace to 300 °C, which made it possible to exclude the presence of biological impurities.

Samples containing *P. syringae* (*Pseudomonas syringae* pv.*syringae* strain 3023) were prepared as described in our previous work [30,31]. The optical density of the cells was monitored by absorbance at 600 nm. The final optical density of the cells in the samples was OD_600_ = 0.02.

Samples were cooled using a Julabo F-25 thermostat (JULABO GmbH, Seelbach, Germany), controlled by a computer program. The temperature-setting accuracy of the thermostat was ±0.1 °C. Cooling proceeded at a rate of 0.225 °C/min (from +10 °C to −17 °C). Sample temperature was monitored using an LTA laboratory thermometer (Termex, St Petersburg, Russia), with a measurement precision of ±0.01 °C. A more detailed description of the experimental setup is provided in our previous work [32,33,34].

### 2.5. Experimental Procedure

First, 1 mL of water was poured into a test tube, and T_f_ was measured at least 5 times to confirm that freezing occurred below −10 °C. Then, a nucleator was added to the same test tube, and the measurements were repeated. Then, the *Rm*AFP was added to the same test tube, and the experiment was repeated. Repeated experiments were performed changing everything, including the test tube.

For each freeze–thaw cycle, the heating rate from −17 °C to +10 °C was 0.225 °C/min. After setting the temperature to +10 °C, the sample was kept for 10 min to ensure complete melting and thermal equilibrium before the next freezing cycle.

To verify the effects of *Rm*AFP, analogous experiments were performed with the GFP protein, which was used as a protein incapable of binding to the ice surface. GFP had no effect on the freezing temperature of the solutions (Figure S2 in Supplementary Materials). In addition, we tested the effect of insoluble metal oxides that are not ice nucleators, as well as different amounts of ice nucleator powders. For example, CuO in amounts of 1.5 mg and 60 mg affects the freezing temperature equally. An example of such studies is shown in [30]. Furthermore, theoretical works [34] confirm that increasing the amount of powders several times cannot significantly increase the nucleation temperature.

Additionally, we performed experiments in which, following the same sequence, instead of a nucleator or protein, we added from 10 to 100 µL of water, Tris buffer, or phosphate buffer at a concentration of 10 mM to the water in the test tube, in order to test the effect of mixing and the presence of small amounts of buffer on the nucleation temperature. In such experiments, we observed no effect on the nucleation temperature.

All samples used in these experiments originated from one protein fraction after protein purification to minimize baseline variability. A total of 2 to 6 independent samples were tested for each substance studied (Table 1). 23 independent samples were analyzed for pure water, 47 considering the data we received earlier [30]. Each individual sample was measured at least 8 times. A more detailed statistical analysis of the nucleation temperatures of pure water is presented in ref. [30]. Because the variances and sample sizes among the groups are unequal, we performed a Welch’s ANOVA followed by Games-Howell multiple comparisons to evaluate intergroup differences without assuming equal variances. Table 1 shows mean freezing temperatures, standard deviations (SD), 95% confidence intervals (CI) and *p*-values.

## 3. Results and Discussion

To investigate the ability of *Rm*AFP to inhibit ice nucleators, we used an experimental approach applied previously by our group and described in detail in several publications [30,31,32,33,34]. The essence of the approach is that the sample temperature is continuously measured during slow cooling in the experimental setup. The setup for these studies comprises a computer-controlled thermostat and a precision thermometer. The thermostat controls the sample temperature at a given rate according to a specified program, while the thermometer continuously records the temperature directly within the sample [30,31,32,33,34]. We acknowledge that isolating the exact timing of initial nucleation relative to the thermal signal represents an important step. However, such analysis requires synchronized advanced techniques (e.g., high-speed cryomicroscopy or high-resolution infrared (IR) thermography) that are beyond our current instrumentation capabilities.

**Figure 2:**
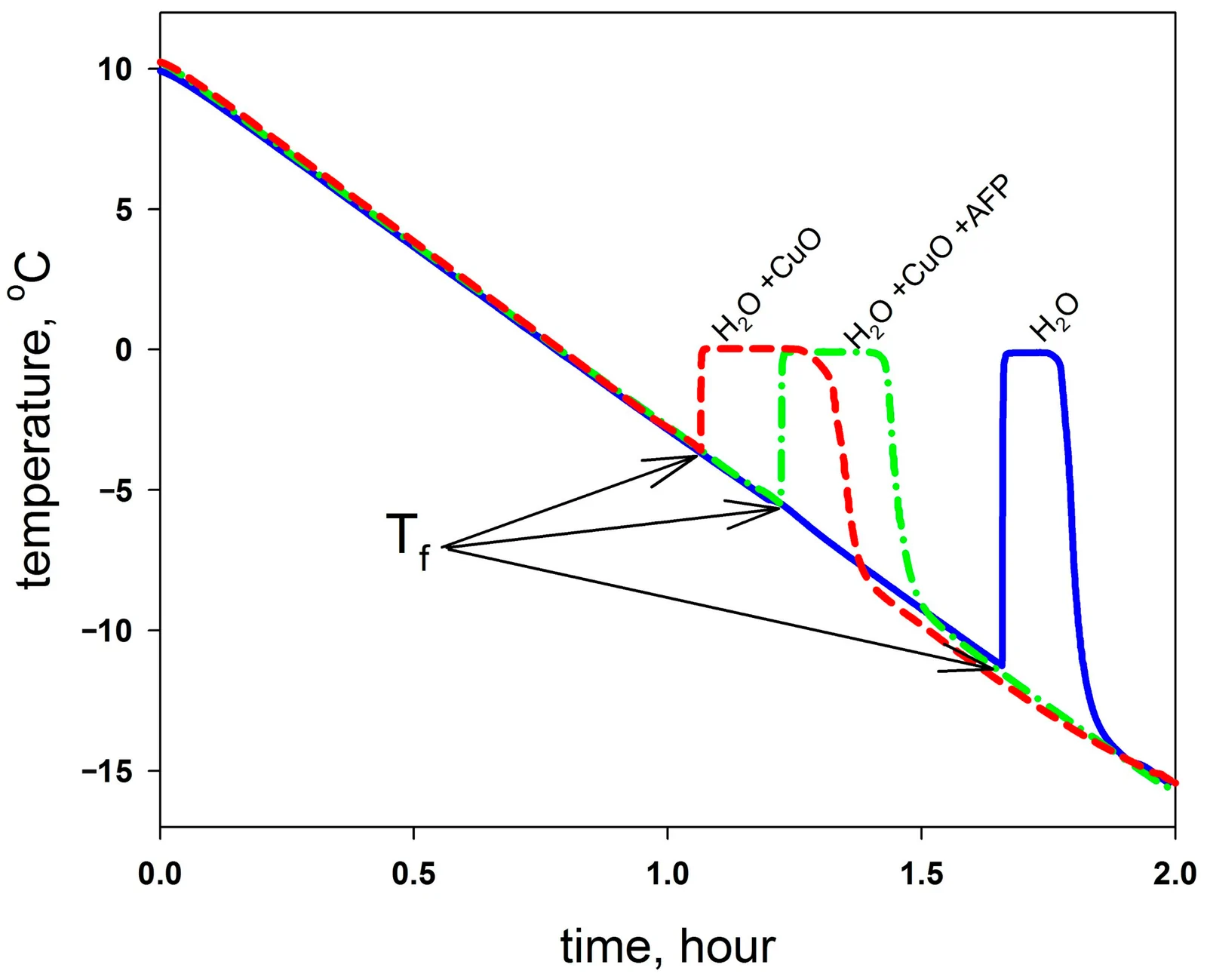
Example dependences of sample temperature on cooling time. Solid blue line—pure water; dashed red line—water containing a nucleator (shown for CuO); dotted green line—water containing a nucleator and *Rm*AFP. The minima on the curves, from which the freezing (nucleation) temperature is determined, are marked with arrows. Cooling proceeded at a rate of 0.225 °C/min (from +10 °C to −17 °C), sample volume was 1 mL.

In such experiments, the moment an ice nucleus forms in the tube is marked by a jump in temperature, since heat is released during ice formation. Figure 2 shows, as an example, typical dependences of sample temperature on cooling time. The minima on the curves, from which T_f_—the freezing temperature—is determined, are marked with arrows.

Such experiments were carried out for pure water, water containing ice nucleators, and water containing ice nucleators together with the *Rm*AFP protein.

**Figure 3:**
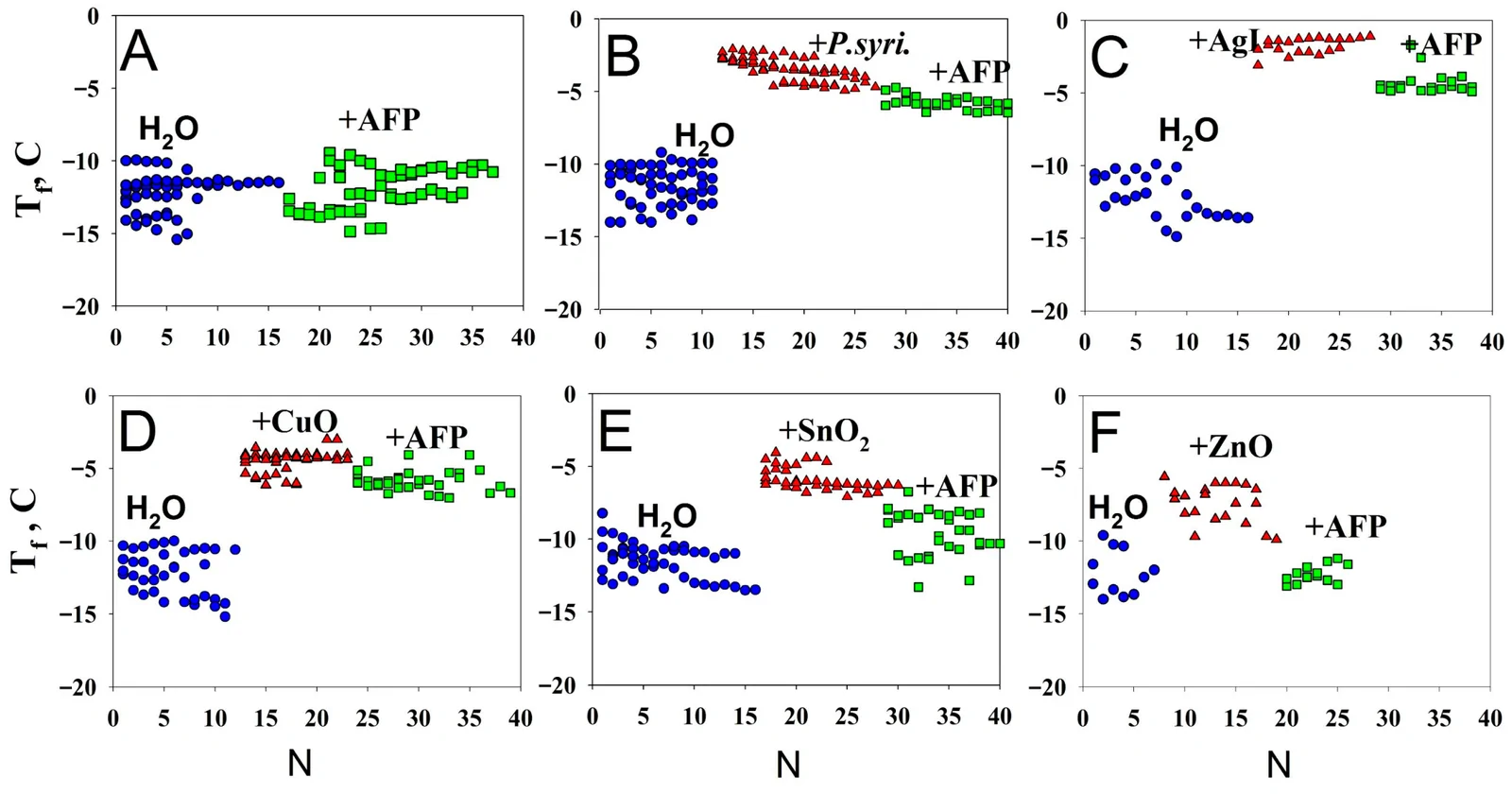
Water freezing temperature in the presence of different nucleators and antifreeze protein. (**A**) for *Rm*AFP only; (**B**) for *P.syringae* and *Rm*AFP; (**C**) for AgI and *Rm*AFP; (**D**) for CuO and *Rm*AFP; (**E**) for SnO_2_ and *Rm*AFP; (**F**) for ZnO and *Rm*AFP; Dependence of the water freezing temperature for water alone (blue circles), water in the presence of nucleators (red triangles), and water with a nucleator and *Rm*AFP protein (green squares). The abscissa (N) shows the experiment number, for ease of visualization. Nucleators are labeled on each panel. The points on the graph that differ in number on the X-axis are obtained as a result of repeated freeze–thaw cycles. Points with the same numbers refer to independent experiments.

As ice nucleators, we chose *P. syringae* bacteria, as well as insoluble powders of AgI, CuO, ZnO, and SnO_2_. We had previously investigated all of these ice nucleators in detail, comparing their effect on the water freezing temperature T_f_ with that of other insoluble metal oxides. Our experiments confirm that *P. syringae* and AgI are the most efficient ice nucleators, triggering water freezing at −3 °C. CuO, ZnO, and SnO_2_ had not previously been described as ice nucleators; their efficiency is lower than that of *P. syringae* and AgI, but still sufficiently high—water freezing in the presence of these substances occurs at temperatures between −4 °C and −7 °C. Figure 3 shows that all of the nucleators increase the water freezing temperature. AgI, CuO, ZnO, and SnO_2_ can currently be considered among the most active inorganic ice nucleators known. Other known nucleators, such as mineral substances, act as nucleators only at temperatures below −10 °C [35,36].

**Figure 4:**
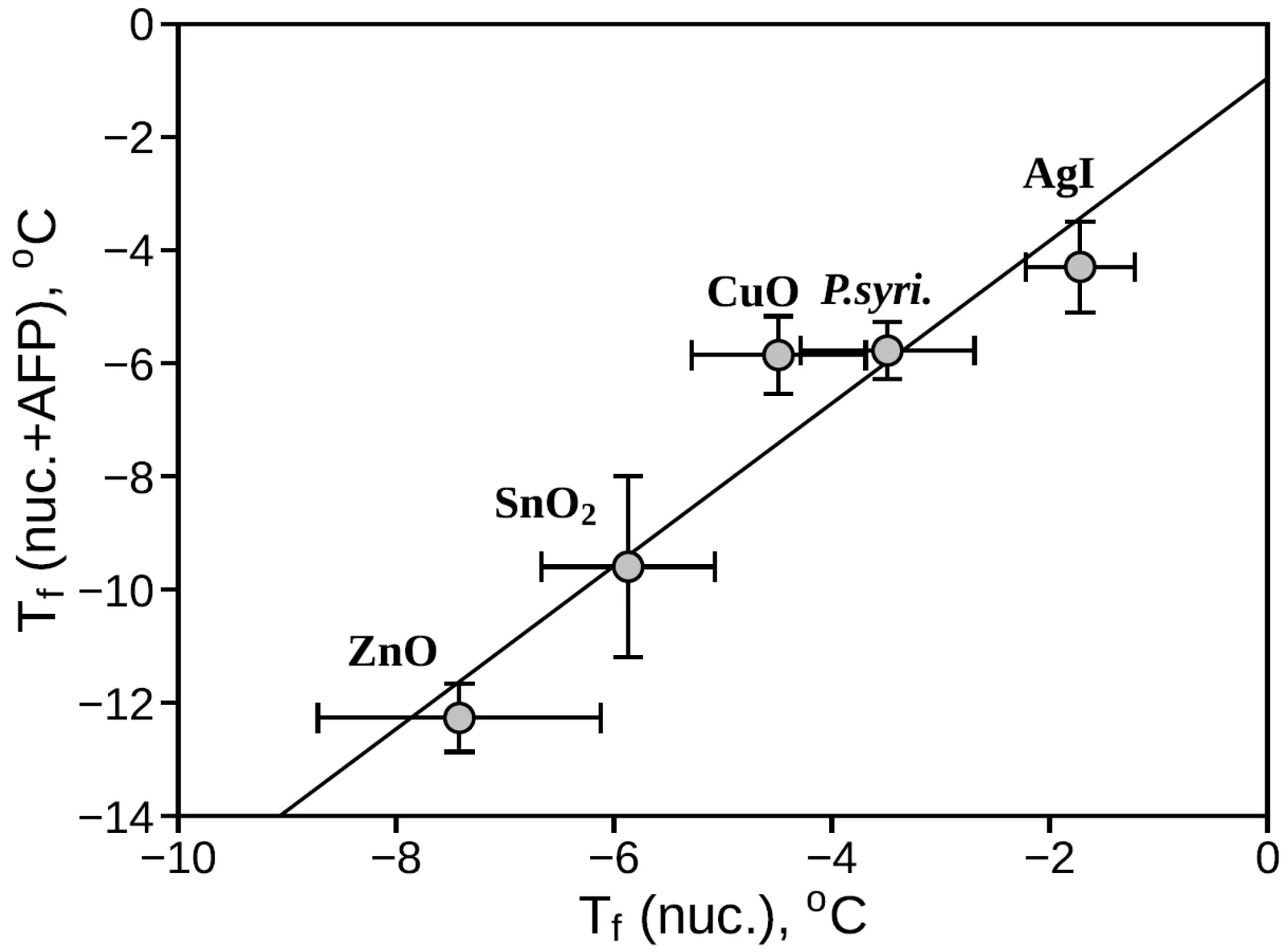
Dependence of the average value of the freezing temperature of water containing a nucleator and *Rm*AFP protein, T_f_(nuc. + AFP), on the average value of freezing temperature of water containing the nucleator alone, T_f_(nuc.). The average values and deviations from the average are calculated from the data in Figure 3. The linear regression in the figure is shown only for convenience of perception of correlation.

Addition of *Rm*AFP to water at a concentration of 0.02 mg/mL does not affect its freezing temperature (Figure 3A), but lowers the freezing temperature of all samples containing ice nucleators (Figure 3B–F). It is evident that the green squares in all panels (Figure 3B–F) lie below the red triangles, indicating that *Rm*AFP blocks the action of all the nucleators tested. Figure 3 also shows that the efficiency with which *Rm*AFP affects different nucleators varies. It is logical to assume that strong nucleators are “harder” to block, while “weak” ones are easier. Figure 4 shows the dependence of the freezing temperature of water containing a nucleator and *Rm*AFP, T_f_(nuc. + AFP), on the freezing temperature of water containing the nucleator alone, T_f_(nuc.). Despite the limited amount of data, a correlation is apparent: the “weaker” the nucleator, the “easier” it is for the protein to block it. A perfect correlation cannot, of course, be expected in such experiments, since the surface structure differs among all the nucleators studied. Nevertheless, the correlation in Figure 4 indicates that the “mechanism” by which *Rm*AFP blocks all of the nucleators is the same. Considering previous work showing that *Rm*AFP can bind to the surface of ice [25,26,27], we suggest that *Rm*AFP “recognizes” the layer of structured water on the surface of any nucleator.

In our opinion, the results obtained allow us to hypothesize that: (1) any nucleator that structures water molecules on its surface can be blocked by *Rm*AFP; (2) antifreeze proteins possessing a face on which several rows of threonine residues structure water molecules (Figure 1) are able to block any ice nucleators. We assume that such a generalization is appropriate, since the nucleators we selected “work” over a wide temperature range from −2 °C almost to −8 °C. In addition, the ability of the *Rm*AFP protein to bind to ice as well as to structure water molecules on its surface [21,22,23,24,25,26,27] suggests not a selective mechanism of binding to specific nucleator surfaces, but a general mechanism via a structured water layer.

One might question whether the number of ice nucleators (INs) and antifreeze proteins (AFPs) tested in this paper (or described in the literature) is sufficient to make the aforementioned generalizations. While broadening the scope of studied proteins (as well as their mutant forms with a reduced ability to block nucleators) and ice nucleators is highly desirable, we faced significant technical and literature constraints that limited further expansion in this study. First, regarding INs, the available options in the literature that are compatible with our experimental setup are extremely limited. Many described INs only initiate freezing at temperatures below −10 °C in highly purified water. This is incompatible with our methodology for two reasons: (i) our ultimate goal is to work with biological cells in buffer solutions rather than pure water; (ii) under standard laboratory conditions, routine purification cannot reliably prevent background water/tube freezing below −10 °C, which would obscure our signal.

As shown in Figure 4, the selected INs trigger freezing uniformly across a distinct temperature range from *T_f_*_(nuc)_ = −2 °C to −8 °C. Testing nucleators with a *T_f_*_(nuc)_ below −8 °C is impractical because our control water samples naturally freeze between −10 °C and −15 °C. Therefore, our selected panel perfectly spans the entire viable window above the background freezing point, providing a robust dataset to generalize the effects of *Rm*AFP across a wide temperature spectrum.

Second, regarding other AFPs, while hundreds have been identified in nature, isolating and purifying them remains highly labor-intensive. More importantly, no clear structural or functional classification system currently exists to guide a representative selection of AFPs for this specific mechanism. *Rm*AFP was chosen deliberately in this study because it is documented as one of the most hyperactive AFPs available. Furthermore, its crystal structure is fully resolved, and existing literature suggests that its activity is driven by a highly ordered, structured water layer on its surface. This makes *Rm*AFP an ideal model to test our core hypothesis that this AFP can serve as a universal inhibitor of ice nucleators before we expand to structurally diverse, less active variants.

The classification of *Rm*AFP as a universal ice nucleation inhibitor remains a well-supported hypothesis rather than a proven absolute. It remains entirely possible that specific nucleators exist that evade its inhibitory mechanism. This hypothesis of universality rests on two primary pillars. First, empirical observations show a distinct lack of substrate selectivity. Contrary to standard ligand-receptor models, where specific binding pairs are expected, *Rm*AFP successfully suppresses every tested nucleator rather than showing a preference for specific chemical compositions. Second, molecular models suggest that *Rm*AFP targets the pre-ordered, structured water layer (the ice-like quasi-liquid layer) organized on the nucleator’s surface, rather than binding directly to the solid chemical interface itself. By recognizing this universal “water fingerprint” rather than the underlying substrate, the protein could theoretically inhibit any ice-nucleating agent.

Because experimentally capturing a stable, direct protein–nucleator complex remains technically unfeasible, this mechanism is supported primarily by indirect thermodynamic data and advanced surface-sensitive analytics. Specifically, heterodyne-detected vibrational sum-frequency generation (HD-VSFG) spectroscopy and molecular dynamics simulations demonstrate that hyperactive insect AFPs, such as *Rm*AFP, possess unique, pre-ordered water domains along their flat ice-binding surfaces [21,23]. These data suggest that *Rm*AFP recognizes the structured water layer on the nucleator’s surface rather than the macromolecular surface itself. When *Rm*AFP approaches an ice-nucleating particle, such as the repetitive TxT-rich matrices of *P. syringae* or inorganic powders, e.g., CuO, the pre-ordered hydration shell of the protein interacts directly with the quasi-crystalline water layers of the nucleator. By forcing a distinct hydrogen-bonding orientation that mismatches the standard ice lattice, *Rm*AFP effectively disrupts the macro-ordering of water required to trigger critical-nucleus crystallization, thereby masking the nucleator site without requiring direct macromolecular contact.

## Figures and Tables

**Table 1:** Analysis of freezing temperatures of samples with various nucleators and nucleators in the presence of *Rm*AFP.

	N Samp.	N	T_f_ Average, °C	SD, °C	*T*-Test *p*-Value
H_2_O	6	50	−12.2	1.4	0.456
AFP	5	46	−11.7	1.4	
*P. syringae*	5	67	−3.5	0.8	0.0034
*P. syringae* + AFP	2	27	−5.8	0.5	
AgI	2	22	−1.7	0.5	0.023
AgI + AFP	2	20	−4.3	0.8	
CuO	4	44	−4.5	0.8	0.032
CuO + AFP	3	33	−5.9	0.7	
SnO_2_	4	38	−5.9	0.8	0.017
SnO_2_ + AFP	3	32	−9.6	1.6	
ZnO	2	21	−7.4	1.3	0.0035
ZnO + AFP	2	13	−12.3	0.6	

N samp.—the number of independent samples (for which everything was changed, including the test tube). N—the number of all nucleation temperature (T_f_) measurements. CI—95% confidence intervals. SD—standard deviation. *p*-values were calculated using Student’s *T*-test with pooled variance. It is assumed that measurements in different test tubes may differ, but equality of variances is also assumed, which is true for stochastic freezing of water. *p* < 0.05 was considered statistically significant. Student’s classic *t*-test showed statistically significant differences. Welch’s *T*-test (which does not assume equality of variances) gave a consistent result. Both variants of the analysis (given in the Supplementary Materials) indicate a statistically significant difference in averages at the selected significance level of 0.05.

## Data Availability

The data presented in this study are available on request from the corresponding author.

## Supplementary Materials

The following supporting information can be downloaded at: https://www.mdpi.com/article/10.3390/polym18212625/s1, Figure S1: Verification of the presence of the antifreeze protein in the cells before and after IPTG induction by SDS-PAGE in a 15% polyacrylamide gel; Figure S2: Testing the effect on CuO of a protein that is not an antifreeze protein (GFP); Table S1: Statistical analysis of the data presented in Figure 3.
