# Supplementary material for: The Ultimate Icebreaker: The Rhagium mordax Antifreeze Protein as a Universal Ice Nucleation Inhibitor
Source: Polymers (Basel). 2026 Oct 23;18(21):2625. doi: 10.3390/polym18212625 (PMC13765640; doi:10.3390/polym18212625)
Supplement: Supplementary file 1 [file polymers-18-02625-s001.zip › polymers-4605156-supplementary.pdf]

Supplementary Materials

The Ultimate Icebreaker: The *Rhagium mordax* Antifreeze Protein as a Universal Ice Nucleation Inhibitor

Maria A. Majorina [1], Vladimir N. Uversky [2,*] and Bogdan S. Melnik [1,*]

[1] Protein Spectroscopy Group, Institute of Protein Research RAS, Institutskaya 4, 142290 Pushchino, Russia; mariamazhorina@yandex.ru
[2] Department of Molecular Medicine and USF Health Byrd Alzheimer's Research Institute, Morsani College of Medicine, University of South Florida, Tampa, FL 33612, USA
* Correspondence: vuversky@usf.edu (V.N.U.); bmelnik@phys.protres.ru (B.S.M.)

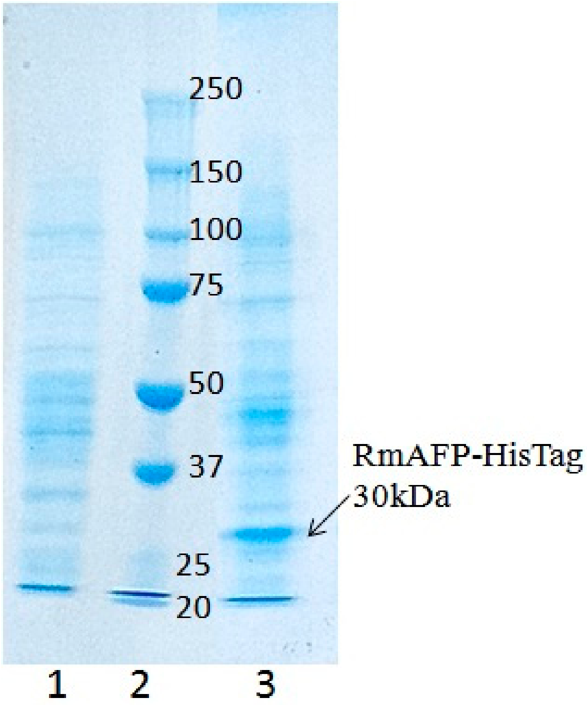

Figure S1. Verification of the presence of the antifreeze protein in the cells before and after IPTG induction by SDS-PAGE in a 15% polyacrylamide gel. Lane 1. *E.coli* with RmAFP-HisTag plasmid in the absence of IPTG. Lane 2. Precision Plus Protein™ Dual Xtra Standards. Lane 3. *E.coli* with RmAFP-HisTag plasmid in the presence of IPTG.

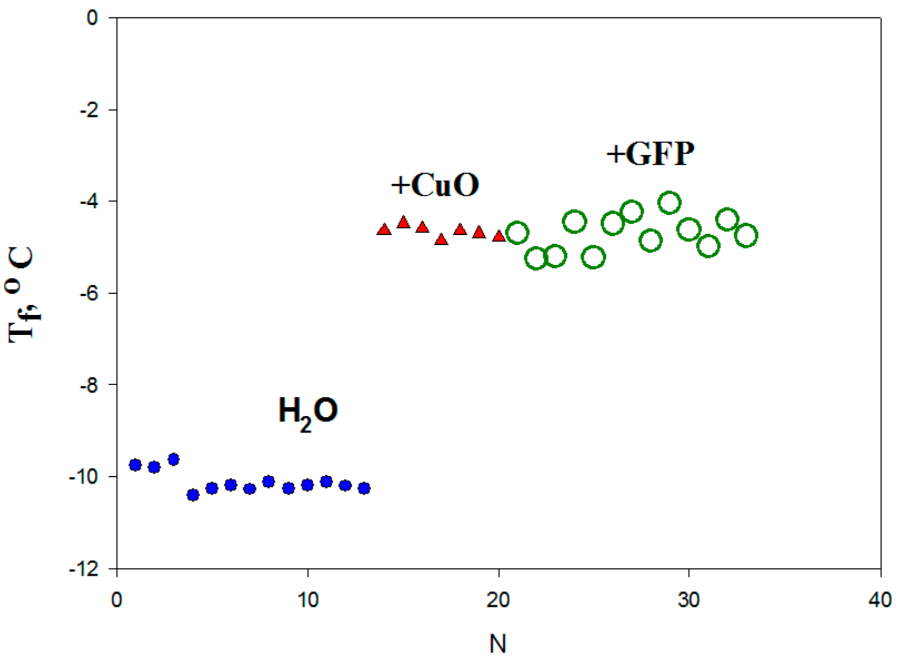

Figure S2. Testing the effect on CuO of a protein that is not an antifreeze protein.

Table S1. Statistical analysis of the data presented in Figure 3.

	**N samp.**	**N total**	**T_f average, °C**	**Δ, °C**	**SD, °C**	**CI, °C**	S. T-test **t**	S. T-test **df**	S. T-test **p**	W. T-test **t**	W. T-test **df**	W. T-test **p**
H2O	6	50	-12.19	1.1	1.4	0.4	0.78	8	0.456	0.76	7.27	0.470
AFP	5	46	-11.67	1.3	1.4	0.4						
CuO	1*	7	-4.67	0.1	0.1	0.1	0.10	5	0.92	0.14	4.98	0.893
CuO+GFP	2	13	-4.71	0.3	0.4	0.2						
P.syri	5	67	-3.48	0.7	0.8	0.2	5.2	5	0.0034	6.18	3.69	0.0045
P.siri+AFP	2	27	-5.78	0.3	0.5	0.2						
AgI	2	22	-1.72	0.5	0.5	0.2	6.42	2	0.023	5.24	1.81	0.042
AgI+AFP	2	20	-4.30	0.3	0.8	0.2						
CuO	4*	44	-4.49	0.5	0.8	0.2	2.77	6	0.032	3.22	5.33	0.021
CuO+AFP	3	33	-5.85	0.5	0.7	0.3						
SnO_2	4	38	-5.87	0.6	0.8	0.3	6.09	5	0.0017	5.16	3.19	0.012
SnO_2+AFP	3	32	-9.59	1.3	1.6	0.6						
ZnO	2	21	-7.42	1.1	1.3	0.6	16.8	2	0.0035	13.75	1.01	0.045
ZnO+AFP	2	13	-12.29	0.5	0.6	0.4						

N samp.—independent samples (for which everything was changed, including the test tube).
N total—the number of all nucleation temperature (T_f) measurements.
CI—95% confidence intervals.
SD—standard deviation.
Δ—the average of the absolute values of the deviations of the data points from the average
p-values were calculated using Welch's t-test. $p<0.05$ was considered statistically significant.
*—taking into account the verification data for GFP (figure S2), 5 independent samples were studied for CuO.
S. T-test—Student's T-test with pooled variance. Assumes equality of variances, which is true for stochastic freezing of water.
W. T-test—Welch's T-test (The Welch criterion does not require equality of variances).
t—value of the t-statistic
df—degrees of freedom (for Welch's test, calculated using the Welch–Satterthwaite formula and therefore fractional)
p—two-sided p-value.
Differences were considered statistically significant at $\alpha = 0.05$. For Welch's test, df may be fractional because the Welch–Satterthwaite approximation is used.

Statistical analysis. Since the data had a nested structure, with 7 to 17 values in each independent experiment, it can be assumed that the individual values are not independent observations. In this case, a t-test can be applied. For each experiment, the mean value was calculated, after which the groups were compared at the level of experimental means. Table 1S shows two versions of the two-sided t-test for independent samples: the classical Student's t-test with a pooled variance estimate, which assumes equal variances (S. T-test in Table 1S), and Welch's t-test (W. T-test in Table 1S), which does not assume equal variances. Degrees of freedom for Welch's test were calculated using the Welch–Satterthwaite formula. The calculations were performed in Excel.
